# Creep Properties and Analysis of Cross Arms’ Materials and Structures in Latticed Transmission Towers: Current Progress and Future Perspectives

**DOI:** 10.3390/ma16041747

**Published:** 2023-02-20

**Authors:** Muhammad Rizal Muhammad Asyraf, Mazlan Rafidah, Emrah Madenci, Yasin Onuralp Özkılıç, Ceyhun Aksoylu, Muhammad Rizal Razman, Zuliskandar Ramli, Sharifah Zarina Syed Zakaria, Tabrej Khan

**Affiliations:** 1 Engineering Design Research Group (EDRG), Faculty of Mechanical Engineering, Universiti Teknologi Malaysia, Johor Bahru 81310, Johor, Malaysia; 2 Centre for Advanced Composite Materials (CACM), Universiti Teknologi Malaysia, Johor Bahru 81310, Johor, Malaysia; 3 Department of Civil Engineering, Faculty of Engineering, Universiti Putra Malaysia, UPM, Serdang 43400, Selangor, Malaysia; 4 Department of Civil Engineering, Necmettin Erbakan University, 42090 Konya, Turkey; 5 Department of Civil Engineering, Konya Technical University, 42130 Konya, Turkey; 6 Research Centre for Sustainability Science and Governance (SGK), Institute for Environment and Development (LESTARI), Universiti Kebangsaan Malaysia, UKM, Bangi 43600, Selangor, Malaysia; 7 Institute of the Malay World and Civilisation (ATMA), Universiti Kebangsaan Malaysia, UKM, Bangi 43600, Selangor, Malaysia; 8 Institute for Environment and Development (LESTARI), Universiti Kebangsaan Malaysia, UKM, Bangi 43600, Selangor, Malaysia; 9 Department of Engineering Management, College of Engineering, Prince Sultan University, Riyadh 11586, Saudi Arabia

**Keywords:** cross arms, latticed transmission tower, creep, cantilever beam, numerical models

## Abstract

Fibre-reinforced polymer (FRP) composites have been selected as an alternative to conventional wooden timber cross arms. The advantages of FRP composites include a high strength-to-weight ratio, lightweight, ease of production, as well as optimal mechanical performance. Since a non-conductive cross arm structure is exposed to constant loading for a very long time, creep is one of the main factors that cause structural failure. In this state, the structure experiences creep deformation, which can result in serviceability problems, stress redistribution, pre-stress loss, and the failure of structural elements. These issues can be resolved by assessing the creep trends and properties of the structure, which can forecast its serviceability and long-term mechanical performance. Hence, the principles, approaches, and characteristics of creep are used to comprehend and analyse the behaviour of wood and composite cantilever structures under long-term loads. The development of appropriate creep methods and approaches to non-conductive cross arm construction is given particular attention in this literature review, including suitable mitigation strategies such as sleeve installation, the addition of bracing systems, and the inclusion of cross arm beams in the core structure. Thus, this article delivers a state-of-the-art review of creep properties, as well as an analysis of non-conductive cross arm structures using experimental approaches. Additionally, this review highlights future developments and progress in cross arm studies.

## 1. Introduction

A power transmission line is composed of continuous power cables lifted by an electrical pylon that is usually constructed in a latticed steel tower to lift the cable above the ground. In power line transmission tower systems, the three major electrical cable types employed are 132 kV, 275 kV, and 500 kV [1]. The peak, boom, cage, tower body, and cross arms of the towers are split into separate parts to build a high-strength transmission tower with superior structural integrity [2]. A crucial part of a transmission tower is the cross arm construction, where the extended arm helps to hold the power wire and insulator. This structural element must be rigid and powerful enough to support the electrical cables and endure dynamic wind loads under various weather conditions (normal and stormy days) [3,4]. The major function of a cross arm is to hold and secure the power lines. Currently, a non-conductive material with lightweight and high-strength properties is used for the cross arm in a suspension tower. A suspended latticed tower’s cross arm is often constructed using high-strength materials, including wood or pultruded glass fibre-reinforced polymer (pGFRP) composite [5,6]. To carry electricity from power generators to substations before it reaches consumers, these towers act as network lines.

Creep is a phenomenon in which a solid substance permanently deforms under ongoing and consistent mechanical loads over an extended period. A material’s creep qualities are used to assess its durability over time and creep life expectancy. Understanding creep is essential as it may jeopardise the dependability and serviceability of the structure. Additionally, creep tends to buckle permanently under mechanical stresses caused by prolonged interaction with high levels of stress, which may lead to structural failure [7,8]. The creep strain-time concept and creep compliance are frequently used to describe creep qualities. To ensure the secure operation of in-service components, it is essential to analyse creep material behaviour [9,10]. Conventional tensile, creep, and compressive specimens are commonly used to experimentally assess creep in bulk materials. Due to resource constraints, it is challenging to create a standard specimen using components that are currently in use [11,12,13]. For instance, it may be difficult to find enough materials for a typical composite cross arm specimen.

Both wooden and pGFRP composite cross arms experience severe creep deformation after a certain service period due to the constant load exerted by the power cable. Creep is a critical issue that shortens the lifespan of cross arms, in addition to exposure to extreme climate conditions and biological attacks. Shear yielding, polymer chain slippage, fibre breakage, void development, and growth are the post-effects of creep [14,15,16]. This is because creep is divided into three stages: primary (fast rate), secondary (steady state), and tertiary (rapid rate to rupture) [17]. Due to strain hardening, the deformation pattern exhibits a decreasing tendency over time throughout the initial stage [18]. When the condition of equilibrium is reached between the rate of recovery and the rate of dislocation creation, the second stage, which precedes this phase, exhibits a creep rate that stays virtually constant [19]. After a creep steady-state situation, the tertiary stage occurs and the creep rate quickly increases until the material breaks [17]. If creep analysis is not taken seriously, the occurrence of creep may result in instantaneous material failure without any warning signs due to continuous exposure to creep’s post-effects.

To forecast a material’s creep data and its behavioural pattern over a long period, extension or stress relaxation is necessary for creep computation and analysis [7]. Changes in displacement (extension) under continual loading contribute to the material’s overall strain. Charts of the constant strain and decreasing stress rate indicate that the total stress would decrease in response to the material’s viscous feature but the total strain would stay constant [8]. Similar to cantilever beams, the material relaxes due to the visco-strain behaviour as it continues to deflect under continuous applied stress. To further analyse the steady-state creep, as well as the elastic and viscoelastic characteristics of the experimental data, numerical creep models, such as the Burger, Findley, and Norton–Bailey models, are used [20]. To provide a sound theoretical framework and backdrop to generalise the material’s long-term behaviour, it is crucial to analyse the visco-elastic behaviour of a particular material. This objective aids in the construction of several analytical models and equations to make the material’s time-dependent behaviour more understandable. To obtain technical information about the impact of a material’s configurations on the integrity of cross arms, various experiments have been conducted. To assess structural and physical performance in this type of instance, Asyraf et al. [21] built a full-scale test rig specifically designed for cross arm assembly. Following the building of the test rig, a number of mechanical experiments were conducted to assess the static and creep characteristics of wooden [20] and pGFRP composite cross arms [22,23,24]. In the early stages of research, many researchers, such as Alhayek et al. [25] and Asyraf et al. [26], characterised the coupon-scale specimens of cross arms in long-term service. The majority of these experiments were conducted using Asyraf et al. [27]’s coupon-scale creep test setup. The most recent developments in the GFRP composite cross arm experiments carried out by various researchers are shown in Table 1.

Regarding the creep response of pultruded composite cross arms in 132 kV transmission towers, no research has yet been published. Furthermore, no study has examined the effects of installing a bracing system in cross arms. The current study aims to discuss and describe the creep response of pultruded composite and wood cross arms at both coupon- and full-scale sizes. Additionally, bracing members are added to cross arms to enhance their resistance to creep and assess their creep response. This article focuses on presenting a critical review of the background, as well as the recent progress and development of studies of cross arms in a 132 kV transmission tower conducted by previous researchers. This state-of-the-art review places particular emphasis on creep performance and analysis techniques of non-conductive cross arm structures and includes perspectives on current research progress.

## 2. Cross Arm Components in Latticed Transmission Towers

### 2.1. Background of Cross Arms and Latticed Transmission Towers

A transmission tower is made up of several components to lift high-voltage cables, a support beam for the transformer, and high-voltage equipment support and racks. In general, transmission towers are widely available in various forms, such as latticed steel towers [21,28,32] and tubular steel monopoles [33]. The typical height of the towers ranges from 15 to 55 m, which highly depends on the minimum ground clearance, length of the suspension insulator, ground-wire location, vertical space between the conductors, and maximum sag of the conductor [7,34]. In Malaysia, there are three types of voltages for the transmission lines of transmission towers, 132 kV, 275 kV, and 500 kV [35,36]. 

Transmission towers are generally categorised into four types, tension, terminal (dead-end), transposition (angle), and suspension. These four categories of transmission towers are combined along the electrical grid to carry electricity. Within the electrical grid, the terminal tower is used to connect the electrical cables from substations and generators to the tension tower. To prevent uneven pressure brought on by line weight and tension, which may lead to the collapse of the entire grid line, the tension tower attaches to the tension cable conductors [37]. To change the physical direction of the electrical grid lines, a transposition tower is implemented in the system, which allows cables to be connected while maintaining adequate clearance for the conductors. In contrast, a suspension tower is used to hold insulators that are horizontally suspended from an electric wire and it primarily functions to supports the conductor’s weight [38].

**Figure 1:**
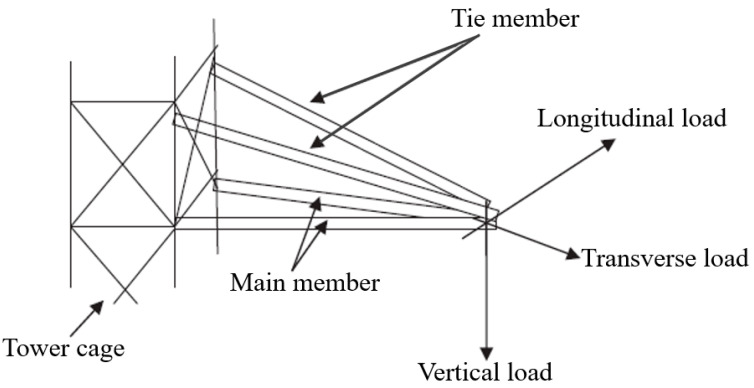
Position of cross-arm components at the cage of the tower (reproduced with copyright permission from Selvaraj et al. [31]).

All transmission towers use cross arm components as extended structure arms that are connected directly to the cage to support the electrical utility cables [39,40]. They are regarded as essential components of the sub-structure of a transmission tower made of latticed steel and they help to keep the insulator and cable attached to the body of the power tower at a specific height above the ground [41]. The cross arms have various shapes and sizes depending on their location and electrical voltage supply. They are also categorised into various sizes, such as 132, 275, and 500 kV. Generally, a cross arm has two types of beams, the main and tie members, which are joined together to hold the cable in both vertical and transverse loads. Figure 1 illustrates the position of the cross arm components at the cage of the tower. 

To keep the costs down while improving the electrical insulation in the surrounding area, cross arms are usually composed of non-conductive materials such as wooden timber or pultruded composites. Overall, the peak, which is situated at the top of a cross arm, is used to guard the earth wire linked to its tip. Additionally, despite being connected to the tower base via the tower body, the cage supports the cross arm.

### 2.2. Materials and Design Structure

Cross arms in suspension towers are commonly made of non-conductive materials to avoid the electrical current from affecting nearby pedestrians. Since the cross arm is used to sustain the cables and wind dynamic loads, they must be made of strong mechanical materials to perform their intended functions. The electrical supply to end users would be disrupted by a failing transmission tower cross arm, which would incur high maintenance costs for electricity providers [36]. Additionally, a faulty cross-arm may increase the risk of injury to passers-by [42]. Thus, it is essential to use strong mechanical material to construct a cross arm. Additionally, the non-conductive materials used in the fabrication of cross arms are required to have a good ability to withstand electrical arcs during lightning strikes. These factors should be considered when selecting the non-conductive materials of cross arms to ensure that the structure can remain in service for more than 24 years [5,28].

Previously, Chengal wood or *Neobalanocarpus heimii* was used as a material for cross arms in transmission towers. It is a tropical heavy hardwood species found only in Peninsular Malaysia [43]. The wood has high mechanical properties, is highly durable, and can be used in heavy structural construction. A comparison of the physico-mechanical properties of Chengal wood and other wood composites for structural application is shown in Table 2. This timber has a fine and uniform texture with a shallow-to-high interlaced grain. It is typically a light-yellow colour that darkens with exposure and its sapwood may turn rust red over time. This wood is considered highly durable, with good resistance to fungi and termite attacks. Tests of Chengal wood showed that treated specimens with dimensions of 50 mm × 50 mm × 600 mm can last around 19 years when subjected to soil burial [44]. In the same test, it was shown that untreated Chengal wood can last only 9 years. Untreated Chengal wood railway sleepers with dimensions of 238 mm × 125 mm × 1950 mm installed under harsh climatic circumstances offer an average service life of 19 years according to another documented study. This study established that Chengal wood is highly durable and has good dimensional stability in extreme environmental conditions over a long period of time.

Recently, pGFRP composites have been commonly used as the cross arm material in transmission towers. The pultruded GFRP composite is fabricated via the pultrusion process. The ratio of E-glass fibre to unsaturated polyester in the pultruded composite is 37:63 [39]. Physically, unsaturated polyester has a density of 1350 kg/m^3^, whereas E-glass fibre has a density of 2580 kg/m^3^. The polymer composite can usually be produced with a fine, uniform, and unidirectional fibre texture throughout the polymer matrix [48,49,50]. pGFRP is laminated with various orientations and densities to maximise its mechanical strength and stiffness. Table 3 displays the recent studies on the influence of the stacking sequence of pGFRP composite cross arms on their mechanical and creep performance. The pGFRP composite with the highest mechanical strength has an optimal sequence of 0°/45°/0°/−45°/0°/−45°/0°/45°/0°, as this sequence provides significant mechanical interlocking of the glass fibres that helps to distribute the excess force.

In general, two materials are currently used in cross-arm applications, Chengal wood and pGFRP composites. Table 4 displays a comparison of the Chengal wood and pGFRP composites used in cross arm structures. Chengal wood has a lower tensile strength and Young’s modulus compared to pGFRP composites. The limited mechanical properties of wood are due to the composition of the natural fibres, which consist of cellulose, hemicellulose, lignin, and pectin [51,52]. This composition results in a weak interaction among the fibres, which promotes internal defects and cracks. Additionally, pGFRP composites are fabricated via a pultrusion process that fully wets the glass fibres to avoid void formation in composite laminates [53]. In terms of physical characteristics, the density of pGFRP composites is relatively the same as Chengal wood and both materials are considered lightweight materials, which allows for the easy installation of the cross arms in transmission towers.

## 3. Creep Properties and Analyses

### 3.1. Creep Phenomenon in Cross Arm Structures

After a successful adaption on a 66 kV tower in 1929, a Chengal wood cross-arm was erected in a 132 kV transmission tower in 1963 [5]. The wood cross arm was chosen due to its exceptional mechanical performance and capacity to extinguish lightning arcs [36]. However, after 24 years of service, the mature Chengal wood showed severe distortion. This occurred because of the natural fibre and wood flaws since the wood was subjected to continuous stress for an extended period. According to earlier sources, it was thus urgent to identify replacements for the wooden cross arms to resolve the problem [39]. pGFRP composite cross arms were installed in transmission towers to replace conventional wood cross arms as an alternative solution. However, ongoing research is being conducted to evaluate the creep behaviour (long-term deformation) of full-scale pGFRP composite cross arms.

Creep occurs when mechanical stress deforms a material over time, causing structural failure [55]. Long-term stress changes a material’s size and form due to creep [56]. Creep analysis estimates the service life of components and products in mechanical and civil engineering [57]. To explain the mechanical phenomenon, creep research uses creep strain and time. Since Hooke’s rule governs creep strain, the two variables are interdependent [19]. The hypothesis states that wood- and FRP-composite viscoelastic materials are elastic until the yield limit. After tension is released, these materials retain the elastic energy and return to their former shapes [58]. When stresses exceed the material’s yield capability, plastic characteristics start to manifest, causing rupture. However, when heated, the materials behave like liquids [59,60]. The general creep stress time comprises three stages: primary (rapid rate creep), secondary (steady-state creep), and tertiary (accelerated rate creep to rupture) [20].

**Figure 2:**
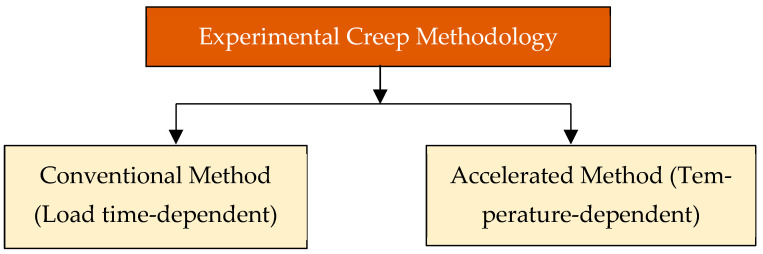
Overview of creep testing methods.

Laboratory tests suggest that it could take a very long time to observe the deformation of polymeric-based materials under certain circumstances. Therefore, the research on creep is impractical since it needs a very long period to collect data. To obtain comparable creep data in less time, experiments are accelerated by exposing the material to a higher temperature [61,62]. This sort of creep testing, known as the accelerated technique, uses heat to accelerate the creep in a shorter amount of time to forecast the material’s lifetime [63]. In particular, the time–temperature superposition principle (TTSP) can be used to illustrate the creep acceleration approach in which many temperatures are shown on a master curve. In this section, both conventional (time-dependent) [64] and accelerated (temperature-dependent) [65] approaches are used to forecast creep behaviour. Figure 2 provides an overview of the approaches used to creep.

In terms of the strain per continuous stress, creep compliance is a parameter that describes long-term durability. *J*(*t*) = (*t*)/0 (where 0 is a constant stress in MPa, (*t*) is the time-based strain under applied constant stress, and *J*(*t*) is the creep compliance in MPa^−1^) can be used to simplify the parameter mathematically. In general, the creep compliance functions are used to uniquely characterise the mechanical properties of a linear viscoelastic material, such as wood or composite, which reflects the growth of strain in a creep test under constant stress [66]. Creep compliance can imitate the true physical behaviour of the material to prevent various questionable or non-physical effects [67].

Creep can be evaluated based on the structural conditions and further analysed using mathematical analysis based on strain–time trends. From this point of view, the long-term durability and serviceability of the structure can be observed under different load percentages prior to the results of the creep compliance test.

### 3.2. Creep Analysis and Properties of Cross Arms: Coupon-Scale Analysis

Research has been conducted on the development of cross arms in latticed transmission towers, including experimental work such as coupon-scale and full-scale analyses. An important first step in characterising a material that has been exposed to real-world variables includes evaluating the loading capacity, temperature, and humidity, which is part of the coupon-scale test [68]. The results of these evaluations can be used as a rough prediction prior to cross arm operations in transmission towers. The coupon-scale test can also be used to carry out a forensic investigation to identify the underlying causes of a collapsed structure in the field of civil engineering. In contrast, full-scale structural research is also necessary to complement the coupon-scale test. To minimise the exaggerated aspects of the coupon-scale test, proper performance tests on long-term creep must be carried out on full-size assembly structures outdoors. During coupon-scale experiments, the structure’s geometrical contour may be disregarded [68]. To understand the true mechanical properties of the composite structure, a full-scale structural test can, therefore, result in more accurate data collection.

**Figure 3:**
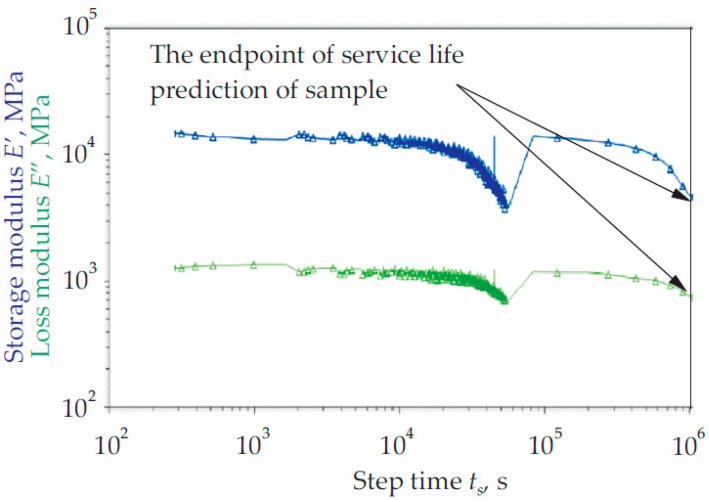
Master curve of both pultruded composite samples at 95 °C, which indicates 25 years of service [69]. Reproduced with copyright permission from Creative Commons Attribution License 3.0.

One of the studies using the creep coupon-sized scale was conducted by Johari et al. [69]. In their experiment, the effects of calcium carbonate on the creep characteristics of a cross arm coupon strip made of pultruded composite that collapsed upon installation in a transmission tower were assessed. Using the time–temperature superposition concept and the traditional bending creep approach for 45 days, they evaluated the creep parameters and predicted the longevity of the composites (TTSP). According to their research on strain deflection (see Figure 3), the material was expected to have a service life of up to 25 years. It was also demonstrated that adding calcium carbonate to a cross-arm composite can increase the composite’s longevity. In addition, it was determined that composite materials both with and without calcium carbonate would still be suitable for use.

Syamsir et al. [70] conducted a study on coupon quasi-static analysis. They investigated how different fibre orientations (0°, 30°, 45°, and 90°) affected the parameters of the composite, including its ultimate tensile strength and Young’s modulus. The study determined that glass fibre composites with three layers and a 0° fibre orientation had the highest ultimate tensile strength and Young’s modulus, measuring 447 MPa and 19 GPa, respectively. In this instance, they concluded that the configuration of the composite exhibited lengthy splitting failure modes, whereas the other configurations showed that the failure was brought on by resin crushing along the fibre orientations of 30°, 45°, and 90°.

**Figure 4:**
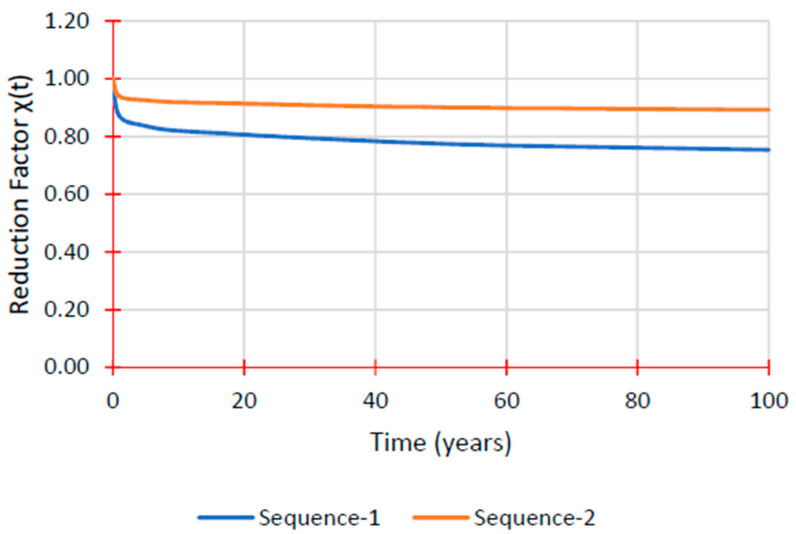
Reduction factor χ(t) prediction over time [25]. Reproduced with copyright permission from Creative Commons Attribution License 3.0.

Alhayek et al. [25] evaluated the creep performance of two stacking sequences of pGFRP composite cross arm coupons under a four-point bending mode. Stacking Sequence 2 (45°/0°/90°/0°/90°/0°) had a substantial flexural strength of 399.9 MPa, whereas Stacking Sequence 1 (45°/90°/0°/45°) had a flexural strength of 242.5 MPa according to the static four-point bending flexural testing. To anticipate the decreased flexural modulus for both sequences, the time-dependent reduction factor (t) was computed at various time intervals. After 50 years, Sequence 1 had a 23% loss, which was substantially different from Sequence 2, which was predicted to lose 10%, as seen in Table 5. These findings, which are shown in Figure 4, demonstrated how the stacking sequences affected the pGFRP material’s characteristics and creep performance.

**Figure 5:**
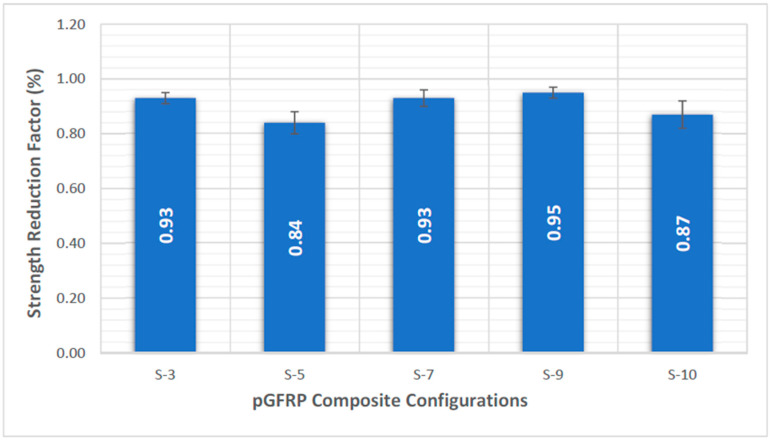
Average strength reduction factor of five different stacking sequences of pGFRP composites over 1440 h (60 days) [71]. Reproduced with copyright permission from Creative Commons Attribution License 3.0.

By analysing different numbers of fibre layers and stacking sequences in terms of quasi-static and creep tests in a four-point bending mode for 1440 h, Asyraf et al. [71] expanded prior investigations. Table 6 displays five different pGFRP composite constructions. The findings demonstrated that nine layers with the following properties had the highest flexural strength: 0°/45°/0°/45°/0°/45°/0°. By alternating between 0° and 45° fibre orientations, this stacking sequence configuration was found to be optimal for continuous roving fibre production. Additionally, as shown in Figure 5, the S-9 pGFRP composite sample had a low creep deflection with strong elastic and apparent creep moduli over a period of 1440 h. This arrangement had the biggest strength decrease factor. The results demonstrated that the nine layers of pGFRP composite with alternating 0° and 45° fibre orientations were well suited for structural use in transmission towers for long-term operation.

**Figure 6:**
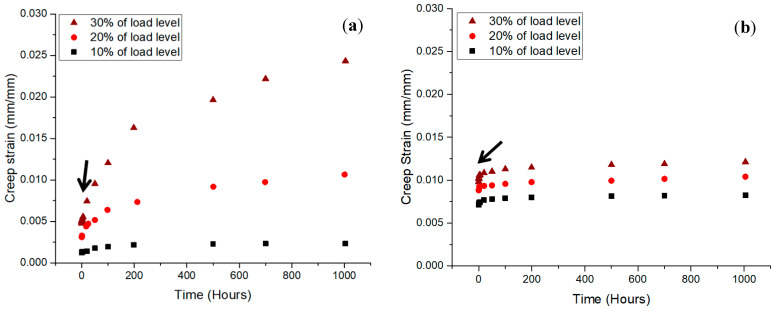
Creep strain against time at different load levels for (**a**) wood and (**b**) pGFRP specimens. Reproduced with copyright permission from Asyraf et al. [26].

The quasi-static and creep performance of wood and composite cross arms at the coupon scale at 10, 20, and 30% load levels for 1000 h was assessed by Asyraf et al. [26]. Figure 6 shows the graphs of the strain time for the wood and pultruded GFRP composite at three different loads. Both the wood timber and pGFRP specimens were found to have creep strains that increased with the load. In this experiment, the two phases, elastic and viscoelastic, were represented by two curves. Sanyang et al. [72] stated that an anisotropic material, such as wood or a polymeric composite, usually acts elastically, then viscoelastically, and then plastically. In this experiment, wood timber took longer to change from an elastic state to a steady viscoelastic state, as shown by the arrows in Figure 6. This shows that the pultruded composite sample had a more stable viscoelastic state than the other materials used for cross arms (wood timber). At the same load level, the force used on the pGFRP laminate was higher than the force used on the wood timber. This is because pGFRP composites with E-glass fibres have better tensile properties [73], and UPE worked well with the glass fibres to ease the stress transfer, leading to better bending performance [74].

**Figure 7:**
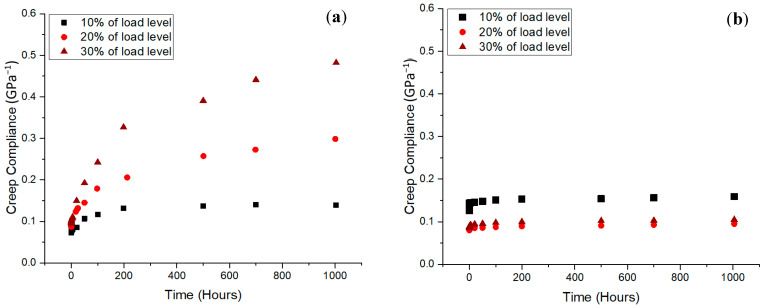
Creep compliance against time at different load levels for (**a**) wood and (**b**) pGFRP specimens. Reproduced with copyright permission from Asyraf et al. [26].

In this research, the authors discovered that the creep compliance results showed that the pGFRP sample had good creep resistance and superior stability moving from the elastic to visco-elastic phases in terms of the creep output. Figure 7 illustrates how the pultruded GFRP has a superior ability to wood for slowing the creep rate, giving the material a longer life under a steady load. The pGFRP specimen had significantly lower creep compliance than wood based on three consecutive load levels. Hoseinzadeh et al. [75] and Nakai et al. [66] found that creep compliance increased due to cell-wall component structural degradation due to daily wet and hot weather. Due to the stress magnitude, micro-cracks propagated between the fibres, causing fibre pull-off [76,77,78]. This was ascribed to the E-glass fibres’ compatibility with synthetic resin (unsaturated polyester) [79], as well as the high tensile properties of the E-glass fibres [80], which prevented the creep from deforming the material continuously during the creep period. As a result, the pGFRP specimen had more bending strength than wood, thus reducing primary creep.

As mentioned by Huang et al. [81], creep failure of beams and structures under long-term service conditions is related to fatigue. These structures are often subjected to cyclic stress, which may reduce their ability to deform under axial stress, worsen their energy dissipation, raise their failure strength, and result in premature hardening [82,83]. According to Movahedi-Rad et al. [84], the creep-fatigue loading pattern with a specific creep time at low stress levels does not influence the fatigue life. Nonetheless, when the degree of stress increases, specimen stiffening occurs during creep loading due to the realignment of the glass fibres, which also lowers the internal friction, hysteresis loop area, and self-generated temperature, hence extending the fatigue life. The restoration of fatigue stiffness is stronger with longer creep duration due to an increase in creep strain, which causes a greater realignment of the fibres. However, the increased creep damage induced by the greater creep strain at high stress levels leads to lower fatigue life. Moreover, it has also been observed that fatigue damage enhances creep deformation.

The results of the numerical analysis showed that the Findley model, which had the least deviations from the experimental results, is the most appropriate creep model for anticipating the creep pattern for both wood and pultruded composite materials. Given their excellent durability, hardwood and pGFRP composites are the most viable options for cross arms. Moreover, the Findley model is the most precise model for analysing the creep characteristics of anisotropic materials. Additionally, under long-term service conditions, the fatigue ability of beams and structures is vital since these structures commonly endure cyclic loads.

### 3.3. Creep Analysis and Properties of Cross Arms: Full-Scale Analysis

The cross-arm structure behaves as a viscoelastic beam, meaning that its stress response decreases over time when the displacement remains fixed at certain locations. This demonstrates how the viscoelastic beam reacts to the viscous properties of the material, gradually reducing the overall stress over time [59,85]. When the material relaxes, the beam continues to deform under a constant load in response to the visco-strain characteristics. It is reasonable to assume that the extension occurring in the top portion of a cantilever beam is tension and that compression occurs in the bottom half of the beam when the beam is analysed for bending. Depending on the thickness and length of a cantilever beam, a non-uniform stress distribution also exists in the beam. Several studies have been conducted on the full-scale analysis of cross arms in latticed transmission towers. In these cases, the 1000 h test was commonly used to evaluate full-scale creep to estimate the creep properties, as well as the lifespan, of the structure. Employing this method can aid in understanding and predicting the long-term mechanical durability of cross arm systems with bracing arms. Additionally, it can offer an intuitive and holistic perspective for assessing the behaviour of the entire structure, effectively facilitating the design of composite products.

**Figure 8:**
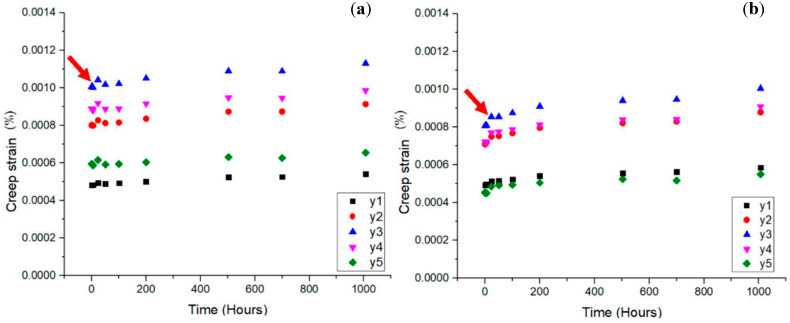
Creep strain against time of right main member of wooden cross arm for (**a**) the current design and (**b**) the design with the addition of a bracing system [23]. Reproduced with copyright permission from Creative Commons Attribution License 3.0.

For instance, Asyraf et al. [23] examined the impact of bracing arms on the creep performance of a wooden cross arm structure in a long-term creep experiment. To simulate the cross arm function in transmission towers, the study used a full-scale cross arm setup with a cantilever beam. The additional support offered by the bracing system greatly increased the creep resistance through a decrease in the creep strain throughout the 1000 h test. They concluded that, as illustrated in Figure 8, the strengthening of the cross arm structure by the bracing arms greatly decreased its creep strain. The cross arm structure’s bracing mechanism improved the wooden structure’s transient creep and stress-independent material properties. The hardwood cross arm structure’s elastic and viscoelastic moduli were also enhanced by the inclusion of the bracing arms. The findings indicated that installing a bracing system in a wooden cross arm can increase the structure’s service life.

**Figure 9:**
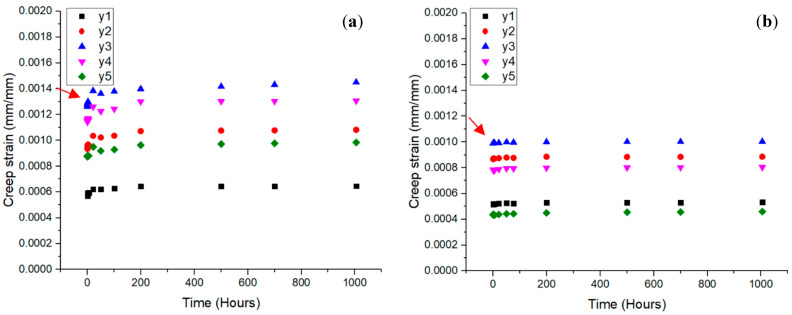
Creep strain against time of right main member of pGFRP composite cross arm for (**a**) the current design and (**b**) the design with the addition of a bracing system [24]. Reproduced with copyright permission from Creative Commons Attribution License 3.0.

Another study carried out by Asyraf et al. [24] employed cross arms made of pultruded glass fibre-reinforced polymer composite. The addition of bracing arms to the structure decreased the overall creep strain on the main member beams of the composite cross arm as shown in Figure 9. The authors also discovered that the elastic and viscous moduli showed increasing trends. The structural integrity and rigidity of the composite cross arm were enhanced by the inclusion of bracing arms, similar to the wooden cross arm. Additionally, the cross arm assembly’s use of bracing arms helped to increase the elastic and viscoelastic moduli for long-term service use.

Based on the previous findings, cross arms must be systematically subjected to creep tests to formally assess and anticipate creep behaviour throughout long-term service. This study compares and evaluates the long-term mechanical performance of the present design used in 132 kV transmission towers to ensure the long-term durability of cross arms. Using a full-sized cross arm in a creep test under real load conditions in an outdoor tropical environment allows for the collection and evaluation of significant data. Table 7 shows a comparison of the creep data and numerical analysis at y3, the middle location of the main member of both wood and pGFRP composite cross arms. Both cross arms exhibited the highest creep strain at the middle location of the main members. All percentage errors were less than 5%, as shown in Table 7. This demonstrated that all the numerical models, including the Burger and Findley models, correctly modelled the experimental data. Zhang et al. [86] stated that a percentage error of less than 20% is acceptable when comparing experimental outcomes with numerical values. When the proportion of errors was under 20%, it was evident that the experimental data that had been plotted did not significantly differ from the guidelines suggested by the precise numerical model. The experimental data that were plotted in this study to elaborate on the creep characteristics of the wooden cross arms were validated with accurate and constant values. In terms of accuracy, the Burger model is more appropriate for analysing full-scale cross arms since the structure exhibits three mechanical conditions, elastic, viscoelastic, and viscous.

From these studies, it can be seen that the elastic modulus of both wooden and pGFRP composite cross arms increases after the addition of bracing arms to the structure. The improvement in the elastic modulus of both brace-enhanced cross arms shows that the addition of the bracing system helped to resist the buckling, as the stiffness of the main member beams increased [87]. In the case of the viscoelastic modulus, the addition of the bracing system to the wooden cross arm structure did not effectively improve its viscoelasticity. In contrast, the viscoelasticity of the pGFRP composite cross arm significantly increased. This shows that the material itself has a better steady-state creep response than wood-based material.

### 3.4. Numerical Methods of Analysing Creep Properties

**Figure 10:**
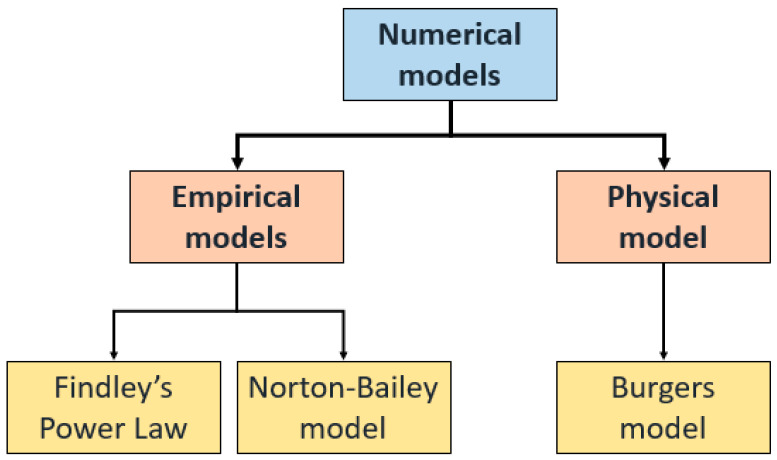
Classification of numerical models for creep analysis.

According to Hao et al. [64], several numerical models have been proposed to calculate a material’s creep qualities. Physical and empirical models are the two categories of numerical models used. The Burger, Findley, and Norton–Bailey models are among the numerical creep models used for calculations of anisotropic materials such as wood and polymer composites. Figure 10 illustrates the classification of these numerical models [7,8,20,88]. The Norton–Bailey and Findley’s power models constitute the empirical models, also known as the power models. The Burger model is the physical model used to interpret creep behaviour. The time-dependent reactions based on strain variations are evaluated using the constitutive models of creep. By establishing the link between the structure and creep behaviour and simulating the creep characteristics of anisotropic materials, the models can determine the creep strain-time trends under constant loads.

#### 3.4.1. Burger Model

**Figure 11:**
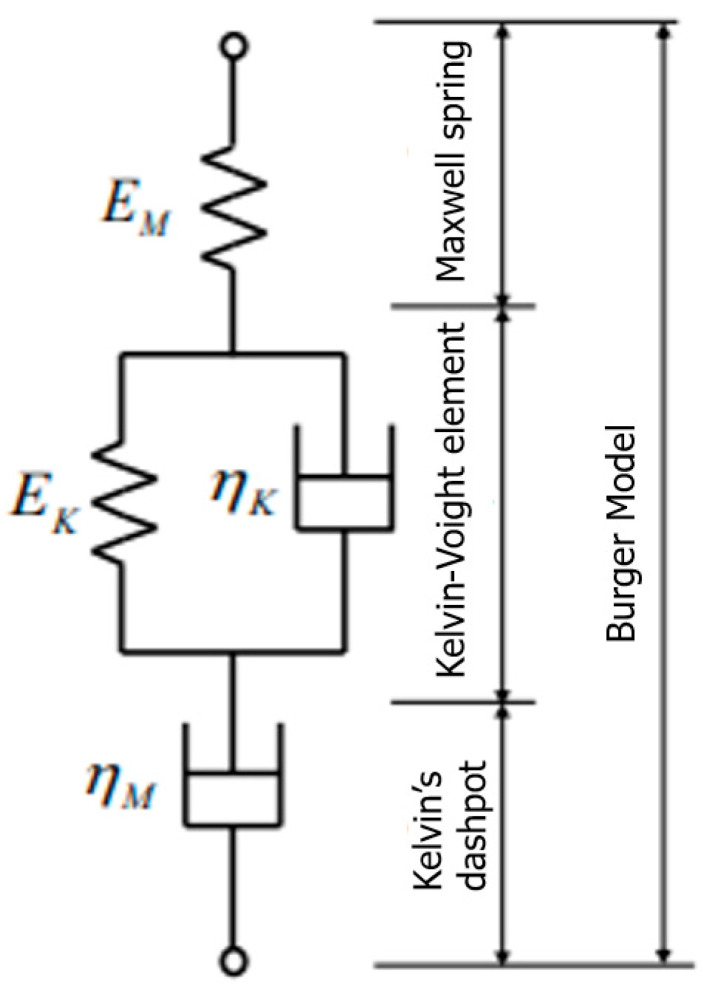
The Burger model comprises a Maxwell spring, Dashpot element, and Kelvin–Voight element [24]. Reproduced with copyright permission from Creative Commons Attribution License 3.0.

The physical Burger model is used to calculate the creep strain-time relationship and assess the elastic, viscoelastic, and permanent deformation qualities. Since the model uses spring and dashpot arrangements to explain the creep phenomenon, it is categorised as a physical model. Materials typically experience three types of strain over a certain period: elastic strain (Maxwell spring), viscoelastic strain (Kelvin’s dashpot element), and viscous strain (Kelvin–Voight element) [89]. According to their time-dependent reactions, the results from the creep experiment using the Burger model can offer a trustworthy creep hypothesis. The Burger model is schematically represented in Figure 11.

The Burger model is one of the models used to determine the physical model-based link between the material and the creep parameters [59]. Equation (1) can be used to represent this model.

(1) $$\begin{array}{c} Total\;strain=Elastic\;strain+Permanent\;strain+Viscoelastic\;strain\\ \varepsilon \left(t\right)=\varepsilon_{e}+\varepsilon_{d}+\varepsilon_{v}\\ \varepsilon \left(t\right)=\frac{\sigma }{E_{e}}+\frac{\sigma }{E_{d}}\left[1-exp\left(-\frac{E_{d}}{\eta_{d}}t\right)\right]+\frac{\sigma }{\eta_{k}}t \end{array}$$

*E_e_*, *E_d_*, *η_d,_* and *η_k_* are all independent variables in Equation (1). Therefore, the model can be described as having four parametric variables. To be more precise, this model can be used to expound on the elastic and viscoelastic moduli, which are both crucial aspects of a material’s behaviour.

According to Hao et al. [64], the Burger model’s simple rheological model can be used to evaluate the creep and recovery responses of kenaf/polypropylene nonwoven composites. Additionally, Moutee et al. [15] employed the Burger model’s global rheological model to evaluate the creep characteristics of a wood cantilever used in wood drying. By employing the Burger model, Wong and Shanks [90] evaluated the creep-recovery properties (elastic deformation, viscoelastic deformation, and viscous flow) of PLA/PHB/flax composites. According to the literature, the model divides the polymer composite into three components: viscoelastic deformation (Kelvin units), viscous deformation, and instantaneous or elastic strain (Maxwell spring) (Maxwell dashpot). In the Burger model, the first term is constant and independent of time; the second term contributes to the early stage of creep, which quickly achieves its maximum; and the third term identifies the long-term creep trend at a constant rate.

#### 3.4.2. Findley Model

The Findley model, also known as Findley’s power law, empirically describes the creep properties using the power-rule method. The model is very useful for predicting the creep strain-time curves using transient creep, which is further clarified using the material stress exponent and stress-dependent coefficient [91]. However, this empirical model is limited in application since the numerical formula is quite straightforward for evaluating creep performance. This is because the model lacks deliberation on the dimensional changes of materials in creep testing [92]. Additionally, the model itself has fewer capabilities and needs to be derived to further analyse the creep properties based on external factors such as moisture content, humidity, and temperature [93]. These external factors contribute to the shape-changing behaviour and deformation of the structure of the material during the creep process.

The model is presented in Equation (2) [94].

(2) $$\begin{array}{c} Total\;strain=Elastic\;strain+Transient\;strain\\ \varepsilon \left(t\right)=\varepsilon_{0}+at^{b} \end{array}$$

Equation (2) comprises the material constant and stress-dependent coefficients, *a* and *b*, respectively. Instantaneous strain is denoted as $\varepsilon_{0}$ after applying the load.

As mentioned by Pérez et al. [95], Findley’s power law is applied as a constitutive model to analyse the creep behaviour of layered silicate/starch–polycaprolactone blend nanocomposites. Furthermore, Fu et al. [20] analysed creep responses and developed a general creep equation for wood floor high-density polyethylene/laminated veneer lumber coextruded composites using the Findley model. Another study by Yang et al. [96] characterised the tensile creep resistance of polyamide 66 nanocomposites using the Findley model. These studies employed the Findley model to evaluate the viscoelastic properties of polymer composites based on instantaneous strain, the amplitude of transient creep strain, and the stress-independent material exponent. The Findley model is suitable for evaluating most anisotropic materials, especially plastic, with good accuracy over a wide time scale and is not sensitive to stress. It is also frequently employed to predict long-term creep properties due to its satisfactory applicability and simple expression.

#### 3.4.3. Norton–Bailey Model

The Norton–Bailey model is an additional empirical model used to assess primary and secondary creep under constant load and temperature conditions during a specified time. In other words, the model describes creep as the relative creep strain and examines the creep strain-time curve only in the secondary creep stage (steady-state creep). This model can also be described as the time-hardening formulation of the power law creep. By studying the creep crack initiation and creep crack growth, the model can also predict the creep damage attributes [97].

The model is expressed by Equation (3).

(3) $$\varepsilon \left(t\right)=m\sigma^{k}t^{n}$$

where *m*, *k*, and *n* are the constant functions of temperature that are generally independent of stress. *A*, *m*, and *n* are three temperature-dependent material parameters that are independent of stress. *n* and *m* are unitless, whereas *A* is the creep strain-hardening coefficient. In this case, *m* is a creep-hardening coefficient that is consistent with the time and stress variables [98].

Mohammadizadeh et al. [99] evaluated the creep properties of nylon reinforced with glass, Kevlar, and carbon fibres manufactured by 3D printing technology using the Norton–Bailey model. In addition, Li et al. [100] conducted a creep study on composite sandwich beams with GFRP face sheets and a balsa wood core using the Norton–Bailey model as a tool to measure their creep response and predict their service life. As mentioned in relation to previous works, the Norton–Bailey model forecasts the strain behaviour of creep of materials exhibiting time-dependent, inelastic strain. 

## 4. Future Development of Cross Arm Structures in Latticed Transmission Towers

New design ideas, new materials, and optimised power delivery with a limited right of way will be needed for future power transmission lines [101]. Lightweight materials that address efficiency and environmental concerns drive power transmission businesses such as those that manufacture cross arms [102,103,104]. FRP composites can replace conventional materials and offer essential applications for a more sustainable society [105,106,107,108]. Due to technological advances, composite cross arms are more commonly used. Most studies of cross arm structures are conducted using numerical simulations to forecast the theoretical data of the structures. Additionally, coupon specimens of both wooden and composite materials are compared and analysed in many investigations to determine their features [109,110,111]. Despite the abundance of research on cross arm coupon testing, investigations of full-scale cross arms are still sparse. Therefore, a mechanical study of a full-scale cross arm is needed to have a comprehensive understanding of its characteristics. Research on full-scale cross arm construction can provide a range of unique technical and performance benefits for both new buildings and existing transmission lines. As a result, utilities are increasingly using this new technology, ushering in a new era of overhead transmissions. Composite cross arms can now replace wooden cross arms in any overhead line installation. Additionally, to ensure a longer lifespan of cross arms, it is recommended that cross arm structures be enhanced by employing bracing arms, incorporating sandwich cores, and installing sleeves. Thus, in this section, the development of creep test rigs for cross arms, as well as the advancements in cross arm technology, is further discussed to highlight the findings of cross arm research.

### 4.1. Creep Test Rigs as Testing Facilities

Mechanical experiments on scale-model cross arm structures are essential to fully understand the creep qualities and provide a more accurate forecast of the service life of a cross arm. A test rig of cross arms of an appropriate size is needed to conduct different mechanical investigations [88]. To measure the bending strain and stress, a creep test is conducted. These characteristics can be used to estimate the cross arm assembly’s lifespan. Additionally, research on the deflection and deformation of the cross arm member components can provide a more intuitive and comprehensive understanding for anticipating the creep behaviour of the entire structure [112]. Test rig performance must be evaluated in relation to factors such as the design, operation, and working conditions. However, there have been no studies on mechanical or creep testing facilities specifically designed for cross arms that are used outdoors at coupon or full scales.

**Figure 12:**
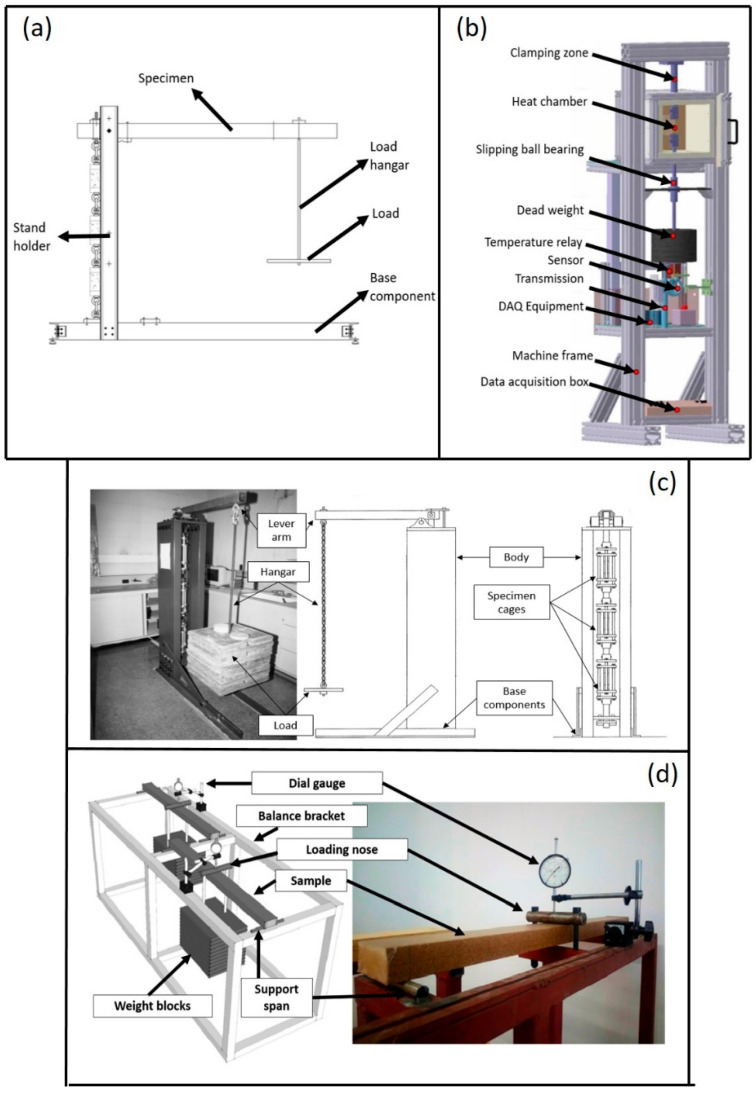
Schematic and overall creep test rig: (**a**) cantilever beam; (**b**) tensile mode; (**c**) compression mode; and (**d**) flexural mode.

Compression, tensile, and flexural modes are the three basic configurations for performing creep tests. In addition, several small-scale experiments have been performed by employing the cantilever beam mode. As a result, several creep test rigs (Figure 12) have been employed by earlier researchers and the outcomes of these tests are as shown below.

Most current creep test rigs focus on coupon-scale testing of either wood or composite specimens. However, a coupon-scale test analysis can only describe the long-term properties of specimens in a controlled environment. In this case, the results of a coupon-scale creep test cannot explain the creep behaviour in terms of structural applications such as cross arms in transmission towers [65,113]. Despite these limitations, a creep study can be conducted in a controlled laboratory environment, which can demonstrate the creep properties under ideal conditions by neglecting other combined environmental factors such as humidity, temperature, sunlight rays, and fungi degradation. Additionally, in these coupon-scale creep experiments, the product shape and possible material changes (inhomogeneities in higher scales) are disregarded, thus providing limited information for making reliable and sound predictions. To accommodate full-scale cross arm components in an outdoor setting, creep test rigs must be built.

To achieve an optimised creep test rig for both coupon- and full-scale tests, product design development must be utilised to ensure design optimisation. To achieve this, the product development process began with an initial investigation by researchers and designers to identify the current problems with existing products. This initial process involved the vital elements of the life-cycle components such as costs, performance, and consumer demands [114]. The data resulting from the initial investigation were used together with a number of software tools such as the material selection tool [115], computer-aided drawing (CAD) [116], and finite element analysis (FEA) [117]. This stage pre-evaluated the initial ideas and proposed several design concepts for the product. In several cases, the integration of engineering approaches and certain tools, such as problem identification tools [118], attribute-refining techniques [119], and design-selection tools [120], provided the optimum qualities and values in the product development process. This method aided in reducing fabrication costs and developing a better-quality, well-functioning product. Overall, design development methods have been widely applied during the product development process, including the Theory of Inventive Problem Solving (TRIZ), the morphological method, and multi-criteria decision making (MCDM).

#### 4.1.1. Theory of Inventive Problem Solving (TRIZ)

**Figure 13:**
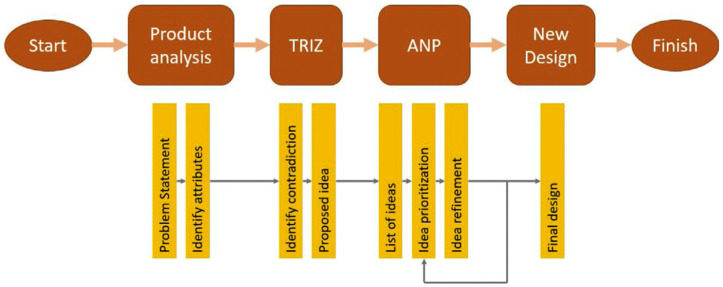
Application of TRIZ in concurrent engineering conceptual design framework for the development of a product [21]. Reproduced with copyright permission from Creative Commons Attribution License 3.0.

The TRIZ is a concurrent engineering technique that can be used to generate design intentions and find solutions to issues by removing any potential trade-offs and focusing on the fundamental causes of the problem [121]. Altshuller (1946) developed the TRIZ tool and divided it into four key approaches. These key strategies include contradiction engineering with 40 creative principles, Su-field modelling, 76 Standard Inventive Solutions, Prediction of Technology Trends, and Algorithms of Inventive Problem Solving (ARIZ) [122]. These methods vary depending on the degree of complexity of the problem being addressed. The systematic approach to solving issues through opportunity identification and problem-solving innovation techniques is very helpful. Cascini et al. [123] claimed that the TRIZ contradiction matrix can be used to design a new concept for sheet-metal snips. The authors chose general solutions from the 40 creative principles by comparing the improving and worsening parameters. Finally, they used a CAD-based design optimisation tool to hone and visualise the design concept. They improved the design concept using the CAD optimisation tool towards the conclusion of the product development phase. The conceptual design method used by Asyraf et al. [21] is shown in Figure 13.

#### 4.1.2. Morphological Chart

**Figure 14:**
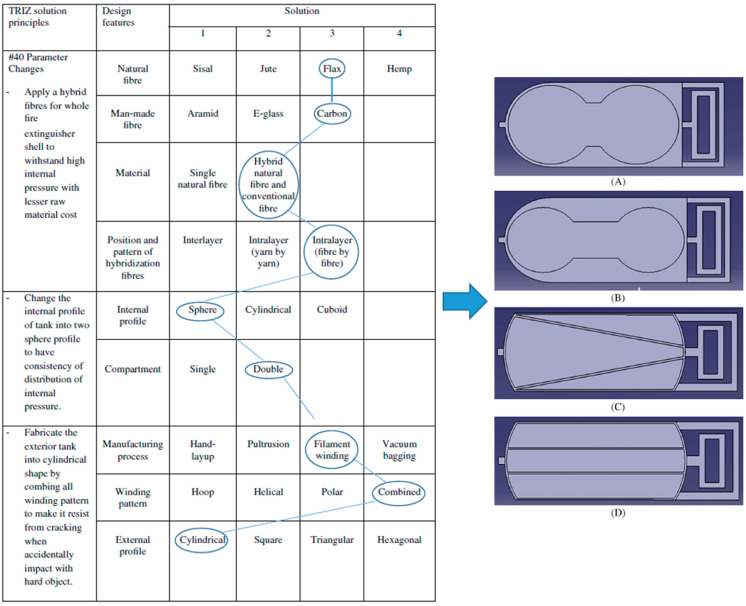
Morphological chart that shows the design features selection of a fire extinguisher prior to development of conceptual designs A, B, C and D. Reproduced with copyright permission from Asyraf et al. [76].

Another concurrent engineering strategy used to create various arrangements is the morphological chart, which enables designers to select novel combinations of components for a product. The term “morphology” refers to the study of shape or form, and the phrase “morphological chart” refers to the summary of methodical efforts to examine the form that a product might use [124,125]. The morphological chart typically assists in providing various possibilities for each element and component that can be combined to create a particular solution. For instance, a study by Sapuan et al. [126] used the morphological chart to produce novel designs for vehicle pedals from polymer composites by combining various design elements through function analysis. The chart provides several options for each part and component, and their combination can produce numerous design features to achieve the product’s functionality. Figure 14 shows an illustration of a morphological chart used to generate and model conceptual ideas for a fire extinguisher made of natural fibre composites [76].

#### 4.1.3. Multi-Criteria Decision Making (MCDM)

Numerous studies and design development projects are carried out by utilising multi-criteria decision making (MCDM) to choose the winning concept design through numerical assessment. The Technique for Order Preference by Similarity to Ideal Solution (TOPSIS) [127], Analytic Network Process (ANP) [128], and *VIseKriterijumska Optimizacija I Kompromisno Resenje* (VIKOR) [129] are three MCDM approaches that can yield a significant solution. Hambali et al. [116] used the ANP approach in the conceptual design selection for composite rubber mounting for vehicle engines. They found that the suggested framework for selection, the AHP, and other concurrent engineering approaches could help designers to control the optimal materials, conceptual design, and manufacturing processes for the product. Through the simultaneous evaluation of many attributes and design choices, these processes can help with design development. During the decision-making process, the suggested concept designs or alternatives can be networked and grouped based on the planned design specifications.

### 4.2. Improvements of Cross Arm Structures in Latticed Transmission Towers

It is essential to overcome the disadvantages related to current cross arms to optimise their long-term durability in 132 kV transmission towers. This may enhance and extend their service life for more than 24 years and indirectly reduce the maintenance costs of transmission towers and, optimally, supply electricity to end-users without disruption [5,130]. Twenty years of operation resulted in the failure of a wooden cross arm structure due to natural wood faults, as reported by the Tenaga Nasional Berhad (TNB) Transmission Division in 2013, which has been the main issue with existing cross arm structures [131]. Therefore, it is crucial to enhance the physical characteristics of the structures, such as installing sleeves, including sandwich structures in the beam profile, or adding a bracing system to the existing structure, to increase the long-term durability or creep-resistant properties of cross arms.

#### 4.2.1. Sleeve Installation

Cross arm structures have an issue regarding creep resistance performance after long-term service. This can cause various problems, especially high maintenance costs and disruptions in the electrical supply to nearby areas. This motivated several researchers to find suitable solutions such as installing a sleeve at the cross arm beams that can function beyond the expected lifespan.

**Figure 15:**
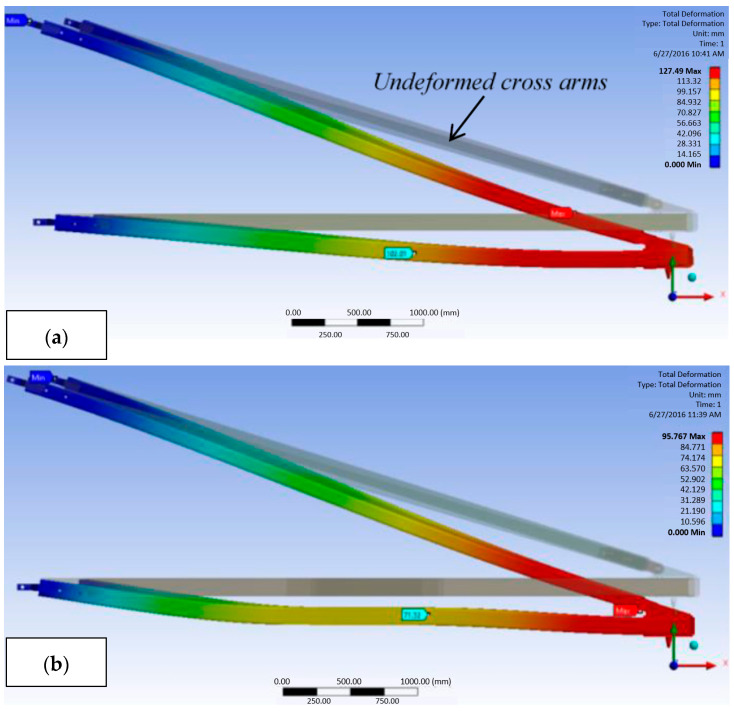
Deformation of (**a**) current composite cross arm, and (**b**) sleeve-enhanced cross arm subjected to multiaxial static loading [2]. Reproduced with copyright permission from Creative Commons Attribution License 3.0.

According to Mohamad et al. [2], adding a one-meter sleeve span to both of the main members of a cross arm can enhance it. ANSYS software can then be used to simulate the structural analysis of the composite cross arm. A one-meter sleeve span is placed on both arms of a cross arm to examine its effects on bending deformation when exposed to multiaxial static stress. The results demonstrated that, as illustrated in Figure 15, the overall deformation of the sleeve-improved cross arm was significantly reduced. A comparison of the deformation outcomes for both the conventional design and the sleeve-improved cross arm is shown in Table 8. Additionally, the stress value of the enhanced cross arm decreased.

In general, the installation of sleeves enhanced the cross arm by lowering the overall deformation once the load was applied. Additionally, cross arms with added sleeves were under less tension. Therefore, the authors recommended installing sleeves as a practical way to increase the lifespan of cross arms and save maintenance costs.

#### 4.2.2. Insertion of Core Material in the pGFRP Cross Arm Beam Profile

Incorporating the core material as a sandwich structure in a cross arm’s beam is another approach to increasing the structural performance under constant long-term loading. Various studies have advocated the use of lightweight core construction to improve structural performance and extend service life.

**Figure 16:**
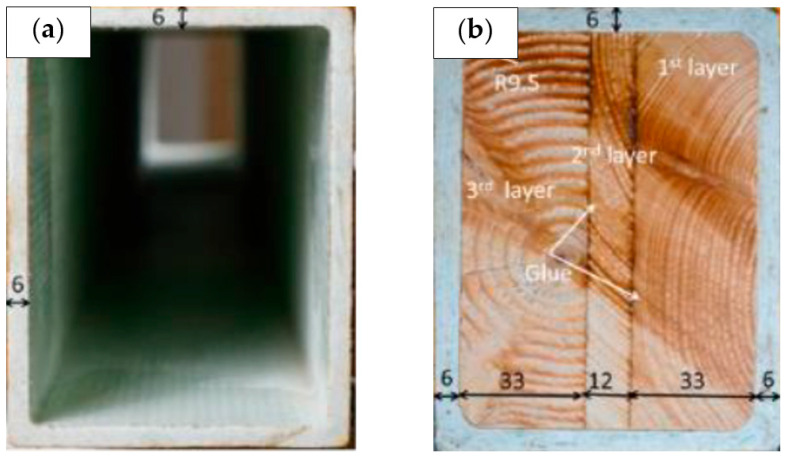
Structural improvement of (**a**) pGFRP square hollow beam by (**b**) insertion of balsa wood core. Reproduced with copyright permission from Zhang et al. [133].

A study led by Qin and Wang [132] demonstrated that the application of a metal foam core sandwich in a square beam structure can increase structural strength. As the total deformation of the local indentation value decreases, the structural strength of the composite core structure greatly improves. This research proves that the use of a core in sandwich beam structures can enhance structural integrity and mechanical performance. In a different investigation conducted using the pultrusion method [133], the authors mounted rectangular wood-cored GFRP sandwich components and discovered that the sandwich portion had the greatest load-carrying ability and a considerable ductility value. This was due to the wood’s lower plastic deformation ability under local compression along the wood grain and sandwich bond, as well as the improved bonding ability of the inner surface of the wood core and pultruded glass fibre composites. According to the research, it is feasible to apply core structures in composite cross arm beams owing to their superior mechanical and structural loading capacity. Figure 16 illustrates the setup used in this investigation.

Honeycomb core is recommended for core structures because of its excellent strength-to-weight ratio [134,135]. Only the core structure is recommended f be incorporated into the primary member beam as it exhibits the most deformation in comparison to other locations. Consequently, the inclusion of core structures in cross arm applications is feasible and can assure the structure’s durability for extended service life and cost savings.

#### 4.2.3. Retrofitting with Addition Bracing Arms

Considering the trusses constructed, such as the ones in transmission towers, there are applications of bracing design that can reduce the occurrence of deformation. To resist the lateral load in steel structures, bracing frames and moment-resisting frames are commonly used. These frames are an effective way to reduce deformation as they apply bracing support to a structure to increase moment resistance [136]. Bracing frames can provide optimal stiffness as they have comparatively lower ductility. On the other hand, moment-resisting frames can contribute to ideal ductility through yielding. However, they do not satisfy the stiffness criteria of most structural applications due to their flexibility. With a combination of both frames, the increased stability and excellent mechanical properties of a steel structure can be achieved [137,138].

**Figure 17:**
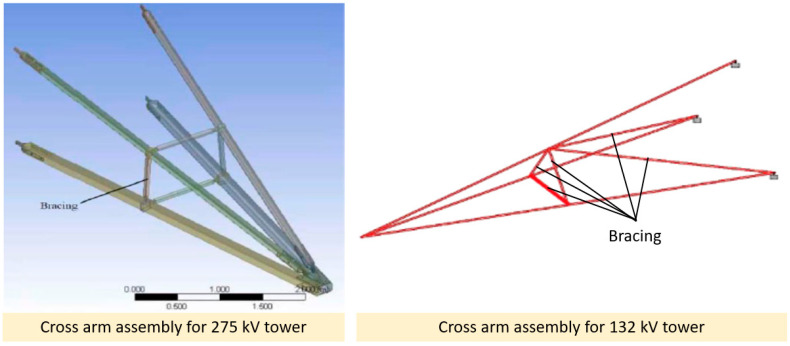
Design improvements of pGFRP composite cross arms in 132 and 275 kV transmission towers by the inclusion of a bracing system [29,140]. Reproduced with copyright permission from Creative Commons Attribution License 3.0.

Another effort that could be considered in the improvement of cross arm structures is to retrofit additional bracing arms. Most structures, such as buildings, bridges, tunnels, and tower cranes, incorporate a bracing system within the structure’s components. The integration of braces into the design of a structural system is very useful for restraining elastic buckling and improving external stiffness [139]. Furthermore, the addition of bracing arms could enhance the axial compressive load-carrying capacity of the structure since the applied external forces are equally distributed between the bracing arms and the main member [29,140]. This approach can also lengthen the service life of cross arms, as well as provide better structural integrity. Figure 17 displays the design improvements of pGFRP composite cross arms in 132 and 275 kV transmission towers by the inclusion of a bracing system.

**Figure 18:**
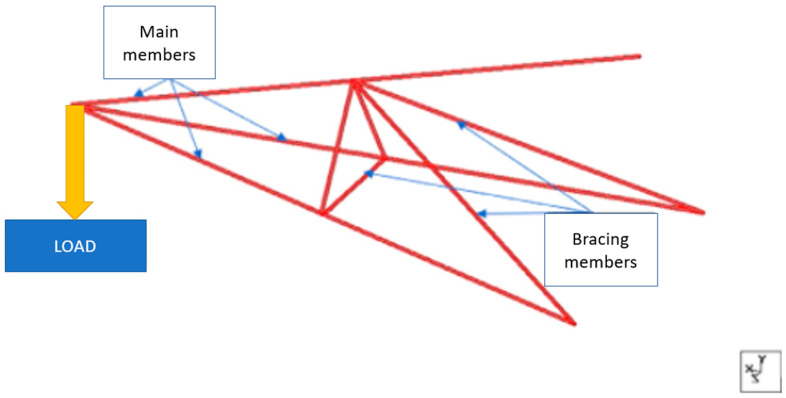
Final design of bracing cross arms in 132 kV transmission tower [29]. Reproduced with copyright permission from Creative Commons Attribution License 3.0.

The study conducted by Sharaf et al. [29] developed a conceptual design of an optimised bracing design for wooden cross arms in 132 kV transmission towers. They applied a hybrid concurrent engineering approach to develop several conceptual designs and simulate those designs using the Skysiv software to characterise them before selecting the final design using ANP. They discovered that the best design was Concept Design 2 (Figure 18) since it exhibited the fewest deflections among all the designs at lower costs.

The current research covers computer simulations to optimise the design of bracing systems to be fitted and installed in current cross arm structures. According to Mohamad et al. [140], the maximum deformation of cross arms in the standard design was 79 mm, whereas after the installation of bracing, the maximum deformation value decreased to 63 mm. This showed that the application of bracing could reduce the maximum deformation of the cross arm by up to 20.3% due to better structural integrity and rigidity. Moreover, the authors also mentioned that the inclusion of bracing support reduced the maximum stress that the cross arms were subjected to by more than 5%. 

Currently, the installation of additional bracing arms as reinforcement for pultruded GFRP composite cross arms in latticed steel suspension towers is still in the trial-and-research stage. The application of bracing arms can enhance the overall structural and long-term mechanical properties of cross arms, as well as improve the durability and service life of pultruded GFRP composite cross arms in a 132 kV transmission tower.

## 5. Conclusions

This work provides an exhaustive analysis of the creep characteristics of non-conductive cross arm materials in latticed transmission towers. In power-grid systems, wood and pultruded composite cross arms are commonly employed in suspension towers to support conductor wires. Nonetheless, several tests indicate that the composite cross arms weaken over time, eventually leading to the collapse of the structure. The structural failure of a composite cross arm can be caused by creep, torsional action, or buckling because of the application of multiaxial loads over an extended period. To counteract this issue, a creep testing technique must be created to characterise creep behaviour and predict the operational lifetime of the structure. Experimental and numerical techniques are used to analyse creep. To be more precise, the experimental methods utilise traditional and accelerated methodologies. Researchers can anticipate the creep of a material using both methodologies to determine its life span. In addition, numerical methods, including physical and empirical models, can be used to validate the experimental work. Both methodologies rely on one another to increase the accuracy of the conclusions and accurately analyse the behaviour of a structure under a long-term steady load. The literature indicates that the composite cross arm has greater creep resistance than the wooden cross arm. Adding calcium carbonate to cross arm composites can also boost the composite’s durability. However, it has been established that both calcium carbonate-containing and calcium carbonate-free composite materials can be used. In addition, a proper stacking sequence and number of layers can considerably enhance a composite cross arm’s creep modulus and mechanical performance. In conclusion, the inclusion of bracing components can improve the creep resistance performance of both wooden and composite cross arms by distributing the concentrated stress throughout the whole assembly.

Although the composite cross arm appears to be more promising than the wooden cross arm, structural improvements, such as the addition of a bracing system, can extend its service life. Thus, cross arm assembly can be improved through the installation of a sleeve, the incorporation of a core into the beam sandwich construction, and the retrofitting of bracing arms into the existing cross arm structure. In addition, action must be taken in terms of the cross arm assembly by analysing the material and overall structure of the cross arm for creep. To achieve these objectives, a creep test rig developed exclusively for both coupon- and full-scale cross arms must be constructed.

## Figures and Tables

**Table 1:** Current research progress of pGFRP composite cross arm studies.

Refs.	Mode of Study	Research	Findings
[21,28]	Cross arm test rig development	Conceptual design of test rig for full-scale cross arms.	- This research used the morphological chart approach to fine-tune the design characteristics and the analytic network procedure to choose designs. It also employed the TRIZ innovative concepts to uncover genuine test rig challenges. In order to build full-scale and coupon-scale cross arm test rigs, concept designs 5 and 3 were chosen.
[27]		Conceptual design of flexural creep test rig.	
[29]	Design of GFRP cross arms	Conceptual design of bracing for composite cross arms.	- This study focused on creating an optimal cross arm assembly bracing design for a 132 kV transmission tower. In order to create an optimal design, this study also employed the hybridisation of the TRIZ–morphological chart–ANP approach. Concept Design 2 was ultimately determined to be the best design to implement in the cross arm framework.
[30]	Experiments	Compressive test for GFRP square-tube columns.	- Rushing, local buckling, and global buckling, which corresponded to each failure, interacted to significantly reduce the capacity of short and intermediate GFRP beam-columns.
[31]		Mechanical tests on pGFRP composite cross arms.	- The axial forces in the main member beams varied linearly with the applied load, with smaller axial forces acting on the tie member of the cross arms.

**Table 2:** Characteristics of Chengal wood in comparison to other wood-based materials.

Material	Density kg/m^3^	Elastic Modulus (GPa)	Rupture Modulus (MPa)	Compression (MPa)		Shear Strength (MPa)	Refs.
				Parallel to Grain	Perpendicular to Grain		
Chengal	915–980	19.60	149.0	75.0	12.0	14.0	[45]
Recycled HDPE/Wood flour composites	900	32.63	40.9	44.97	43.91	27.36	[46]
Laminated veneer lumber	590	6.06	55.0	-	-	14.14	[47]

**Table 3:** Fibre orientation and thickness of pGFRP composite cross arms in previous studies [2].

pGFRP Composite Cross Arm Fabric Orientation (°)	Ultimate Flexural Strength (MPa)	Refs.
45°/0°/45°	267.88	[13]
45°/−45°/90°/0°/45°	175.21	
45°/−45°/0°/90°/0°/90°/0°	355.96	
0°/45°/0°/−45°/0°/−45°/0°/45°/0°	436.29	
45°/−45°/0°/0°/0°/0°/0°/0°/−45°/45°	289.07	
±45°/90°/0°/±45°	242.60	[14]
±45°/0°/90°/0°/90°/0°	399.05	
45°/−45°/90°/0°/45°	421.35	[27]

**Table 4:** Comparison of characteristics of Chengal wood and pGFRP composites as non-conductive materials of cross arms.

Materials	Density (kg/m^3^)	Texture	Tensile Strength (MPa)	Young’s Modulus (GPa)	Rupture Modulus (MPa)	Refs.
Chengal Wood	915–980	Fine and even with deeply interlocked grain	149.00	19.6	149.0	[54]
pGFRP Composites	850–1155	Fine, homogenous, and unidirectional fibre along the matrix	429	34.0	858.0	[1]

**Table 5:** Predicted reduction factor and flexural modulus for each sequence. Reproduced from ref. [25].

Time (Years)	Sequence 1		Sequence 2	
	χ(t)	E(t) (MPa)	χ(t)	E(t) (MPa)
1	0.87	15,619.1	0.94	20,624.1
5	0.84	15,000.3	0.93	20,323.7
10	0.82	14,695.2	0.92	20,176.0
50	0.77	13,893.2	0.90	19,785.4

**Table 6:** Five configurations of pGFRP composite cross arms. Reproduced from Ref. [71].

No.	Configuration	Number of Layers	Layering Sequence
1.	S-3	3	45°/0°/45°
2.	S-5	5	45°/−45°/90°/0°/45°
3.	S-7	7	45°/−45°/0°/90°/0°/90°/0°
4.	S-9	9	0°/45°/0°/−45°/0°/−45°/0°/45°/0°
5.	S-10	10	45°/−45°/0°/0°/0°/0°/0°/0°/−45°/45°

**Table 7:** Creep properties of wooden and pGFRP composite cross arms at the middle main member, y3.

Material	Cross Arm Design	Main Arm	Experimental	Findley Model	Percentage Error (%)	Burger Model	Percentage Error (%)	Elastic Modulus, *E_e_* (10^10^ Pa)	Viscoelastic Modulus, *η_k_* (10^14^ Pa)	Refs
Wooden Cross Arm	Current	Right	1.006	1.010	0.398	1.010	0.398	6.54	6.15	
		Left	0.988	0.994	0.604	0.986	0.202	6.70	3.86	[23]
	Braced	Right	0.806	0.798	0.993	0.806	0.000	8.20	5.37	
		Left	0.731	0.722	1.231	0.756	3.420	8.74	3.50	
Composite Cross Arm	Current	Right	1.262	1.190	6.050	1.310	3.664	18.09	14.10	
		Left	0.996	0.963	3.427	1.010	1.386	23.47	28.06	[24]
	Braced	Right	0.990	0.987	0.304	0.993	0.304	23.87	192.10	
		Left	1.053	1.050	0.286	1.050	0.286	22.58	155.40	

**Table 8:** Deformation results of both composite cross arm configurations. Reproduce from Ref. [2].

Configuration	Mid-Span Deformation (Mm)	Peak Deformation (Mm)
Current design	102.01	127.49
Sleeve-enhanced design	71.32	95.37
Percentage reduction with sleeve installation	30.09%	25.19%

## Data Availability

No data were used to support this study.
